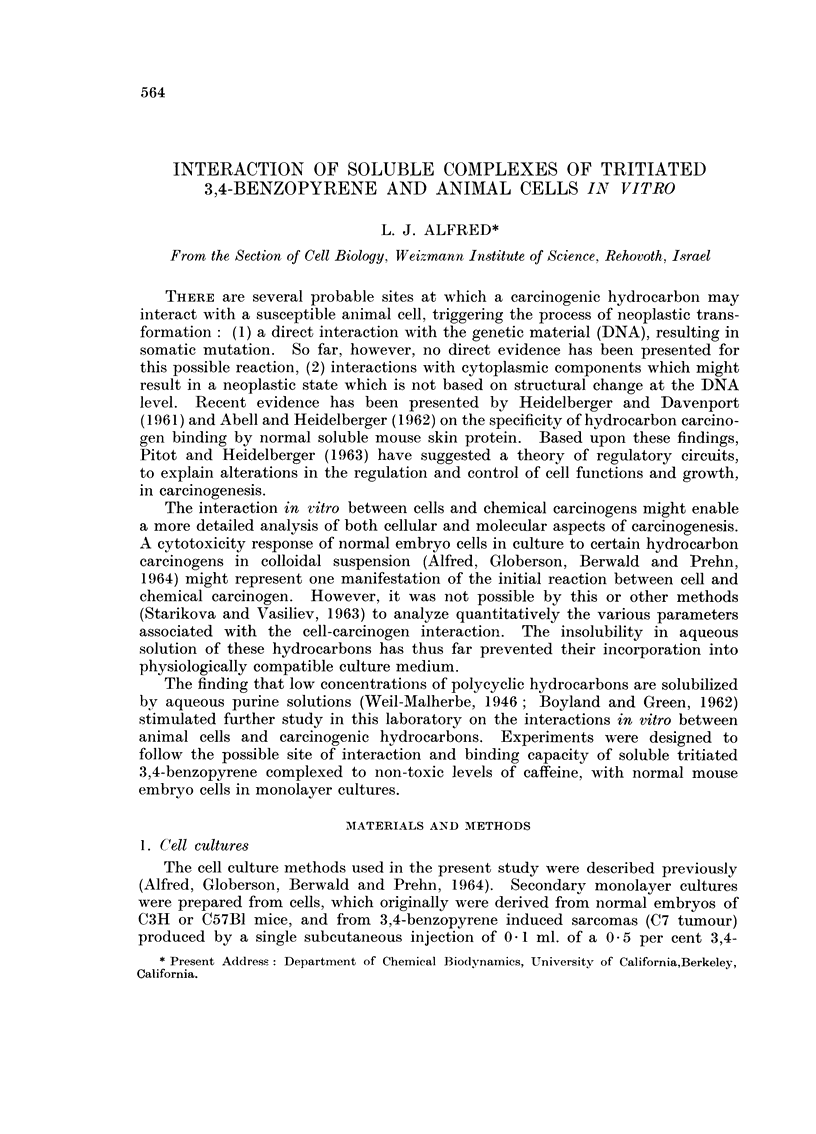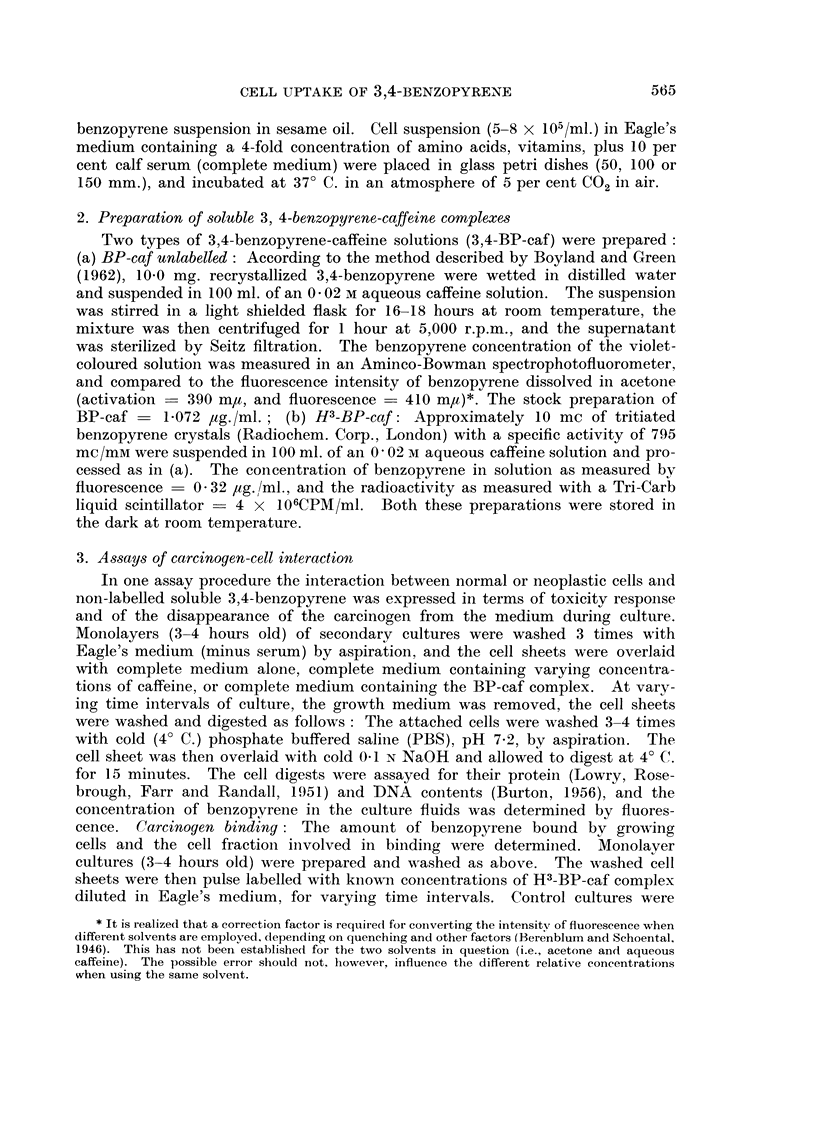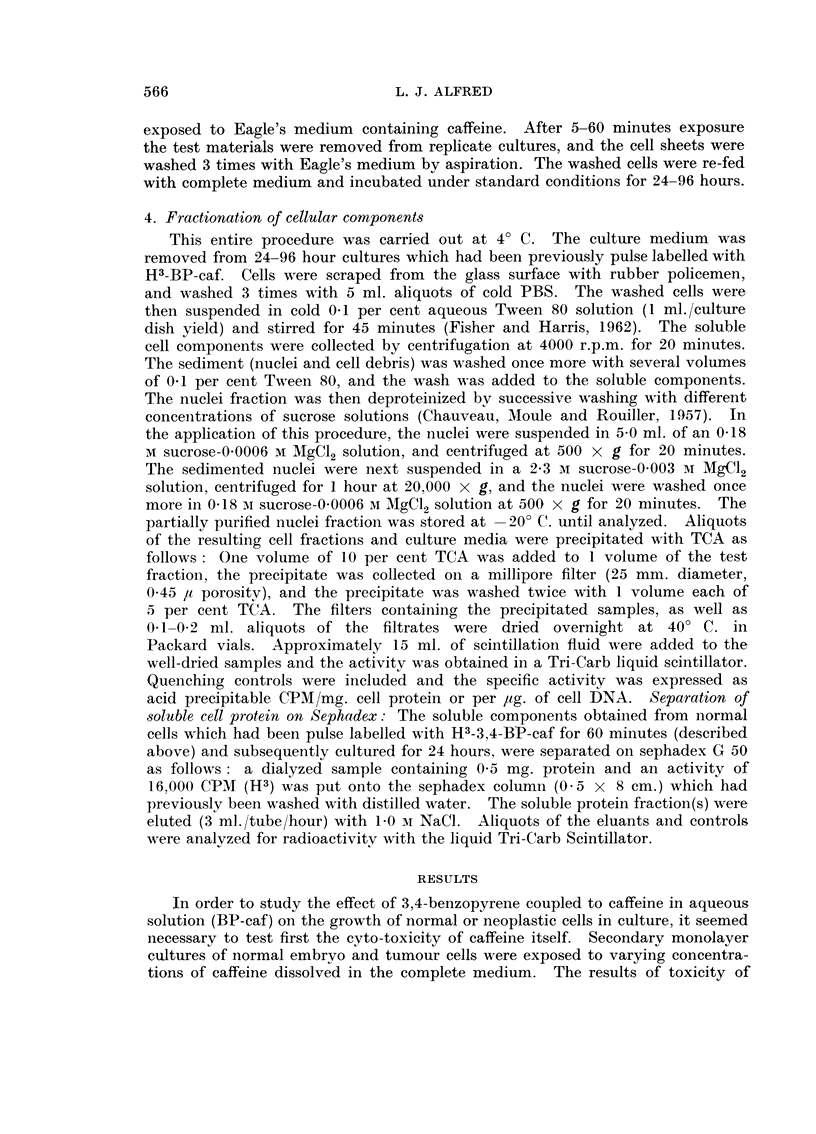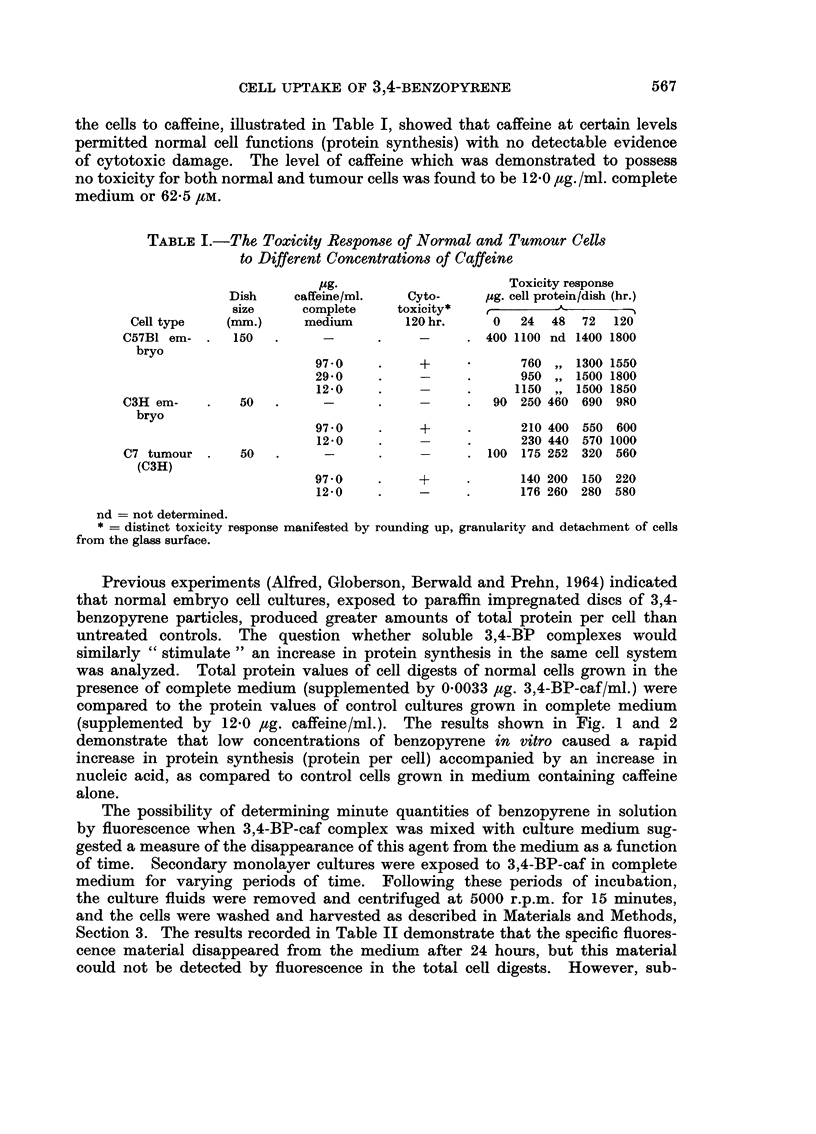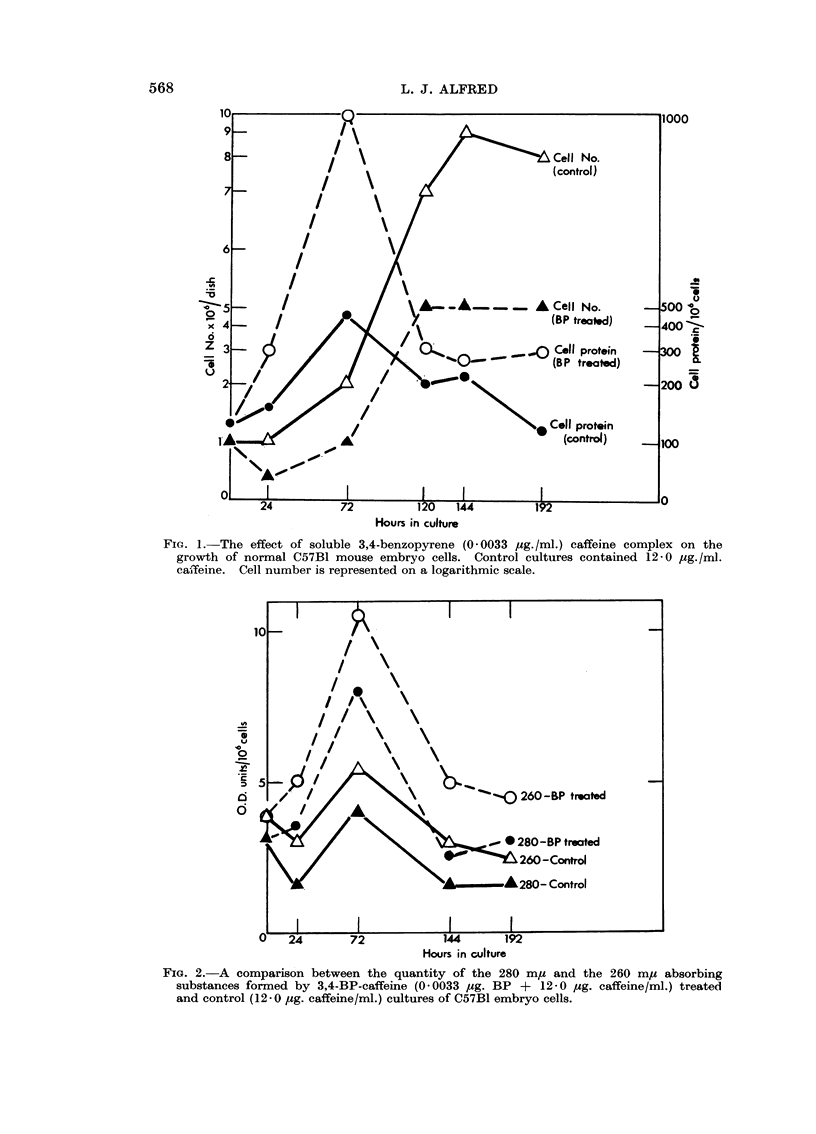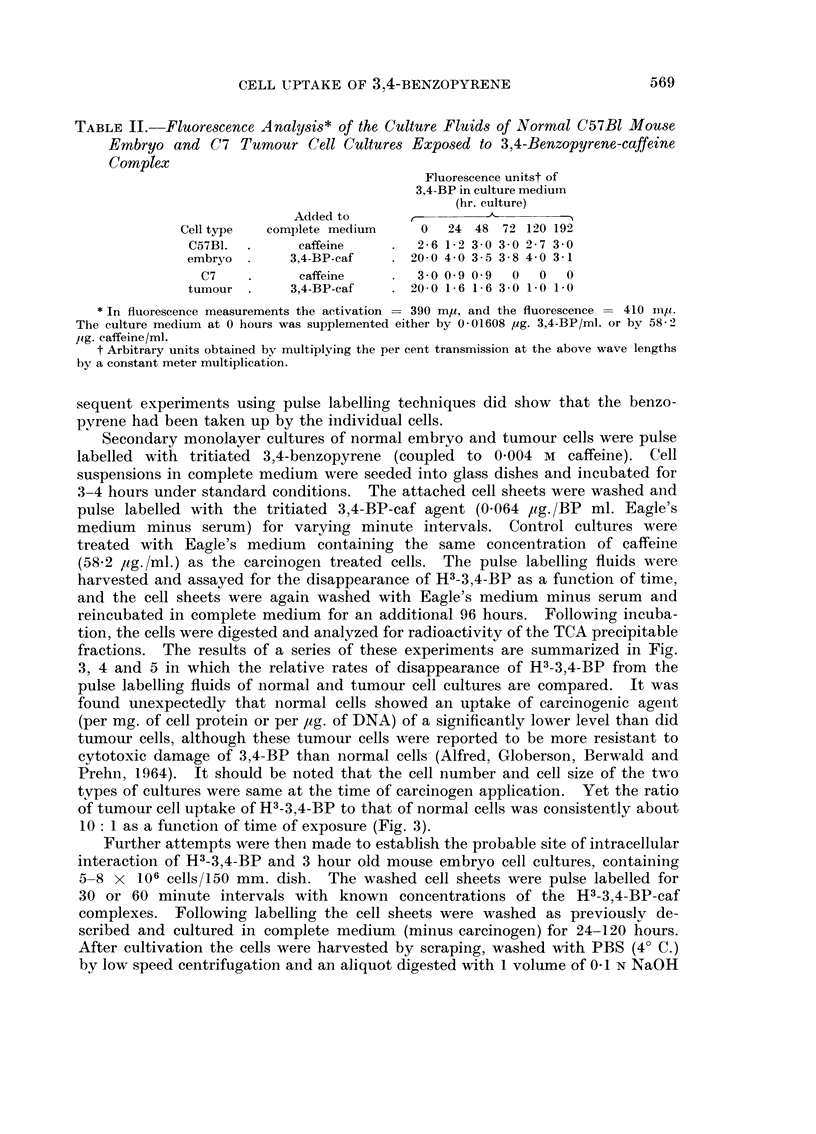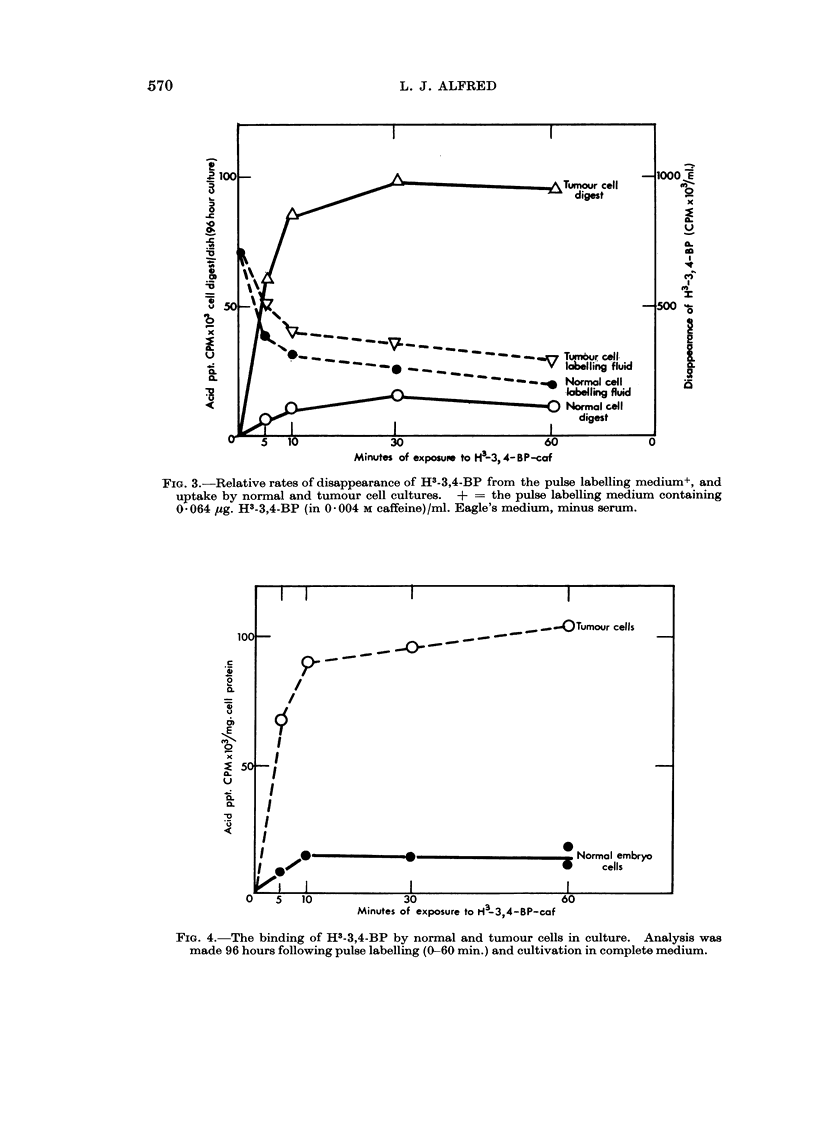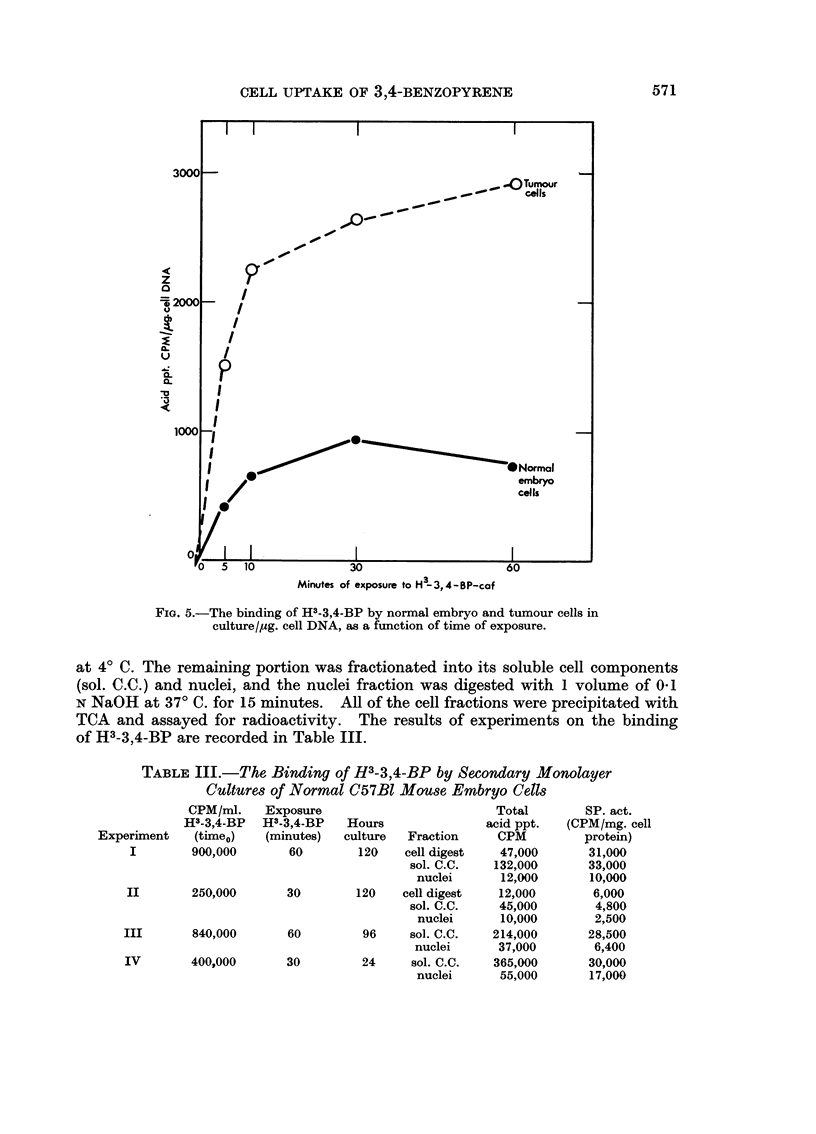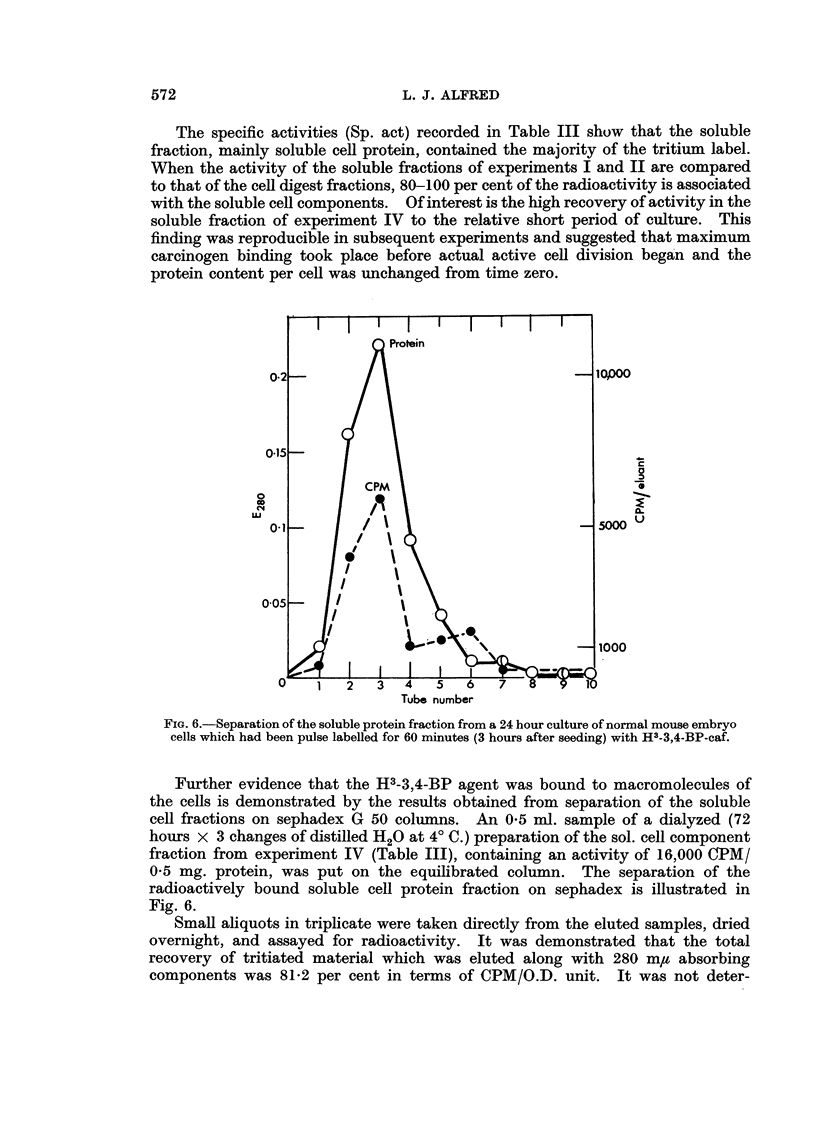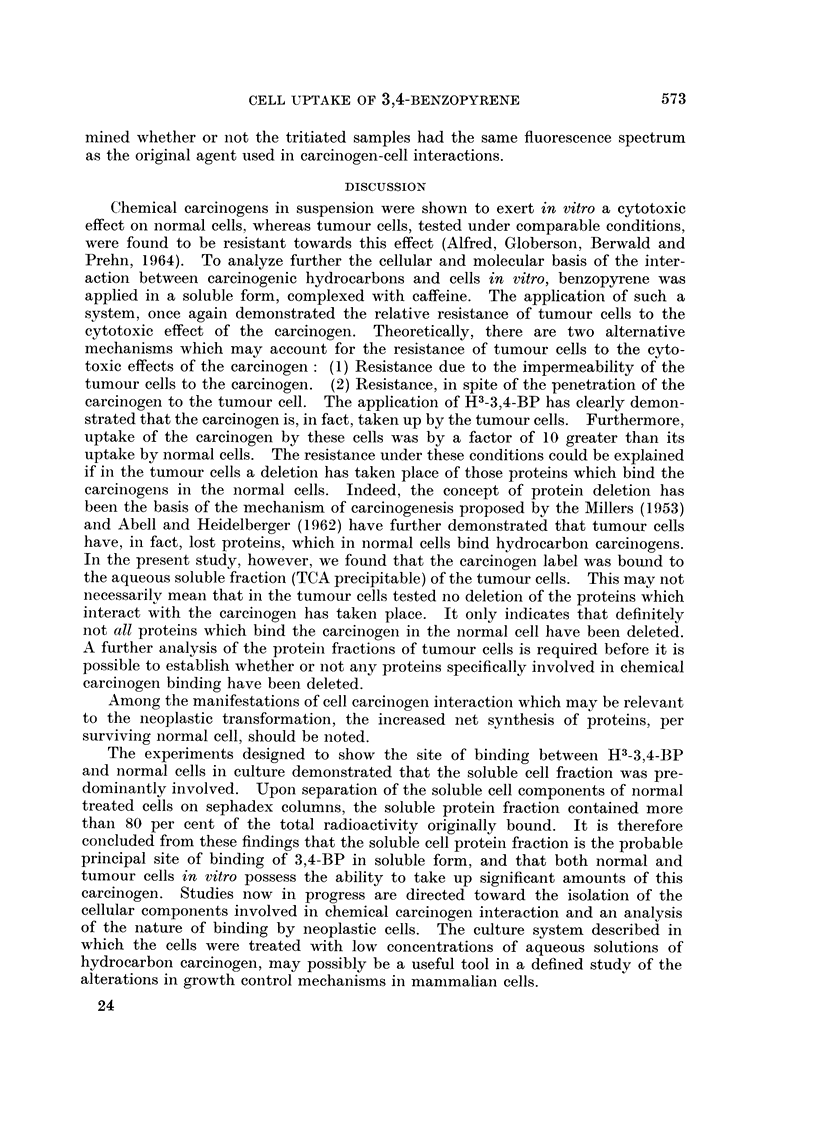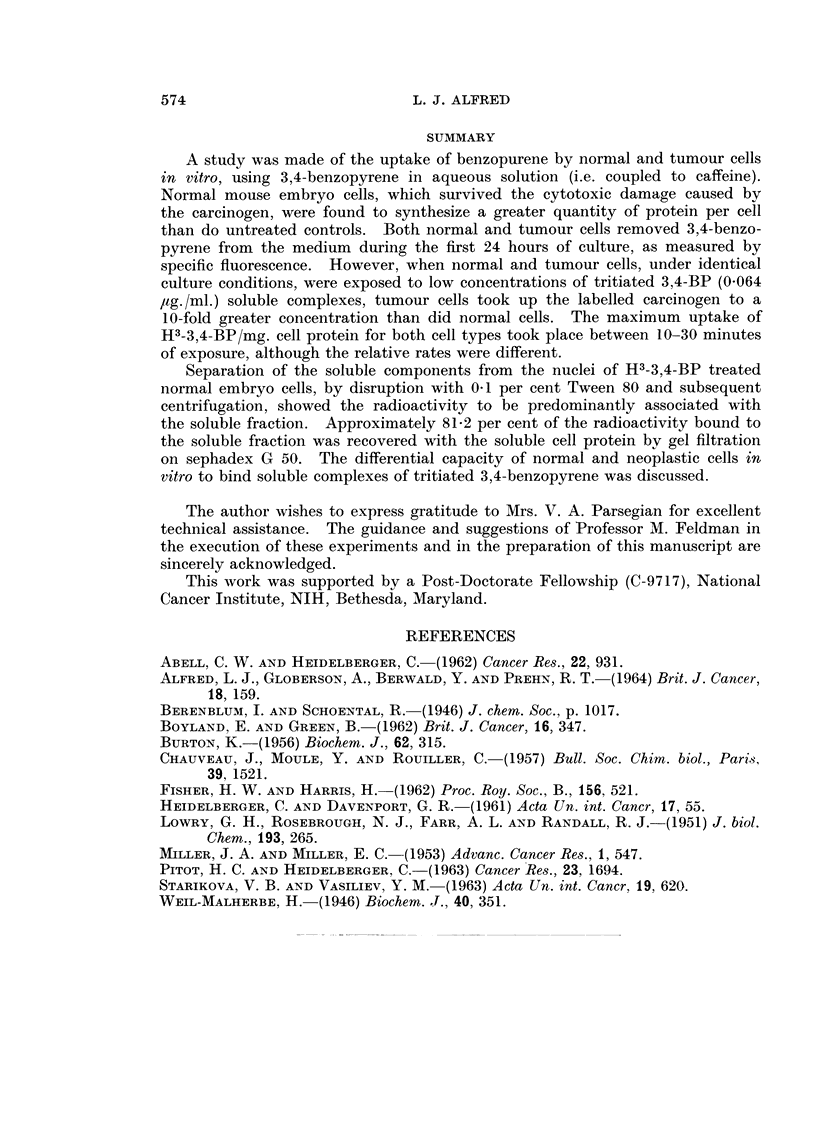# Interaction of Soluble Complexes of Tritiated 3,4-Benzopyrene and Animal Cells In Vitro

**DOI:** 10.1038/bjc.1964.65

**Published:** 1964-09

**Authors:** L. J. Alfred


### INTERACTION OF SOLUBLE COMPLEXES OF TRITIATED 3,4-BENZOPYRENE AND ANIMAL CELLS *IN VITRO*

L. J. ALFRED*

*From the Section of Cell Biology, Weizmann Institute of Science, Rehovoth, Israel*

THERE are several probable sites at which a carcinogenic hydrocarbon may interact with a susceptible animal cell, triggering the process of neoplastic transformation : (1) a direct interaction with the genetic material (DNA), resulting in somatic mutation. So far, however, no direct evidence has been presented for this possible reaction, (2) interactions with cytoplasmic components which might result in a neoplastic state which is not based on structural change at the DNA level. Recent evidence has been presented by Heidelberger and Davenport (1961) and Abell and Heidelberger (1962) on the specificity of hydrocarbon carcinogen binding by normal soluble mouse skin protein. Based upon these findings, Pitot and Heidelberger (1963) have suggested a theory of regulatory circuits, to explain alterations in the regulation and control of cell functions and growth, in carcinogenesis.

The interaction *in vitro* between cells and chemical carcinogens might enable a more detailed analysis of both cellular and molecular aspects of carcinogenesis. A cytotoxicity response of normal embryo cells in culture to certain hydrocarbon carcinogens in colloidal suspension (Alfred, Globerson, Berwald and Prehn, 1964) might represent one manifestation of the initial reaction between cell and chemical carcinogen. However, it was not possible by this or other methods (Starikova and Vasiliev, 1963) to analyze quantitatively the various parameters associated with the cell-carcinogen interaction. The insolubility in aqueous solution of these hydrocarbons has thus far prevented their incorporation into physiologically compatible culture medium.

The finding that low concentrations of polycyclic hydrocarbons are solubilized by aqueous purine solutions (Weil-Malherbe, 1946 ; Boyland and Green, 1962) stimulated further study in this laboratory on the interactions *in vitro* between animal cells and carcinogenic hydrocarbons. Experiments were designed to follow the possible site of interaction and binding capacity of soluble tritiated 3,4-benzopyrene complexed to non-toxic levels of caffeine, with normal mouse embryo cells in monolayer cultures.

### MATERIALS AND METHODS

### 1. *Cell cultures*

The cell culture methods used in the present study were described previously (Alfred, Globerson, Berwald and Prehn, 1964). Secondary monolayer cultures were prepared from cells, which originally were derived from normal embryos of C3H or C57Bl mice, and from 3,4-benzopyrene induced sarcomas (C7 tumour) produced by a single subcutaneous injection of 0·1 ml. of a 0·5 per cent 3,4-

* Present Address : Department of Chemical Biodynamics, University of California,Berkeley, California.

benzopyrene suspension in sesame oil. Cell suspension ($5–8 \times 10^5$/ml.) in Eagle's medium containing a 4-fold concentration of amino acids, vitamins, plus 10 per cent calf serum (complete medium) were placed in glass petri dishes (50, 100 or 150 mm.), and incubated at 37° C. in an atmosphere of 5 per cent $CO_2$ in air.

2. *Preparation of soluble* 3, 4-*benzopyrene-caffeine complexes*

Two types of 3,4-benzopyrene-caffeine solutions (3,4-BP-caf) were prepared : (a) *BP-caf unlabelled* : According to the method described by Boyland and Green (1962), 10·0 mg. recrystallized 3,4-benzopyrene were wetted in distilled water and suspended in 100 ml. of an 0·02 M aqueous caffeine solution. The suspension was stirred in a light shielded flask for 16–18 hours at room temperature, the mixture was then centrifuged for 1 hour at 5,000 r.p.m., and the supernatant was sterilized by Seitz filtration. The benzopyrene concentration of the violet-coloured solution was measured in an Aminco-Bowman spectrophotofluorometer, and compared to the fluorescence intensity of benzopyrene dissolved in acetone (activation = 390 m$\mu$, and fluorescence = 410 m$\mu$)*. The stock preparation of BP-caf = 1·072 $\mu$g./ml. ; (b) *$H^3$-BP-caf*: Approximately 10 mc of tritiated benzopyrene crystals (Radiochem. Corp., London) with a specific activity of 795 mc/mM were suspended in 100 ml. of an 0·02 M aqueous caffeine solution and processed as in (a). The concentration of benzopyrene in solution as measured by fluorescence = 0·32 $\mu$g./ml., and the radioactivity as measured with a Tri-Carb liquid scintillator = $4 \times 10^6$CPM/ml. Both these preparations were stored in the dark at room temperature.

3. *Assays of carcinogen-cell interaction*

In one assay procedure the interaction between normal or neoplastic cells and non-labelled soluble 3,4-benzopyrene was expressed in terms of toxicity response and of the disappearance of the carcinogen from the medium during culture. Monolayers (3–4 hours old) of secondary cultures were washed 3 times with Eagle's medium (minus serum) by aspiration, and the cell sheets were overlaid with complete medium alone, complete medium containing varying concentrations of caffeine, or complete medium containing the BP-caf complex. At varying time intervals of culture, the growth medium was removed, the cell sheets were washed and digested as follows : The attached cells were washed 3–4 times with cold (4° C.) phosphate buffered saline (PBS), pH 7·2, by aspiration. The cell sheet was then overlaid with cold 0·1 N NaOH and allowed to digest at 4° C. for 15 minutes. The cell digests were assayed for their protein (Lowry, Rosebrough, Farr and Randall, 1951) and DNA contents (Burton, 1956), and the concentration of benzopyrene in the culture fluids was determined by fluorescence. *Carcinogen binding* : The amount of benzopyrene bound by growing cells and the cell fraction involved in binding were determined. Monolayer cultures (3–4 hours old) were prepared and washed as above. The washed cell sheets were then pulse labelled with known concentrations of $H^3$-BP-caf complex diluted in Eagle's medium, for varying time intervals. Control cultures were

* It is realized that a correction factor is required for converting the intensity of fluorescence when different solvents are employed, depending on quenching and other factors (Berenblum and Schoental, 1946). This has not been established for the two solvents in question (i.e., acetone and aqueous caffeine). The possible error should not, however, influence the different relative concentrations when using the same solvent.

exposed to Eagle's medium containing caffeine. After 5–60 minutes exposure the test materials were removed from replicate cultures, and the cell sheets were washed 3 times with Eagle's medium by aspiration. The washed cells were re-fed with complete medium and incubated under standard conditions for 24–96 hours.

4. *Fractionation of cellular components*

This entire procedure was carried out at 4° C. The culture medium was removed from 24–96 hour cultures which had been previously pulse labelled with $H^3$-BP-caf. Cells were scraped from the glass surface with rubber policemen, and washed 3 times with 5 ml. aliquots of cold PBS. The washed cells were then suspended in cold 0·1 per cent aqueous Tween 80 solution (1 ml./culture dish yield) and stirred for 45 minutes (Fisher and Harris, 1962). The soluble cell components were collected by centrifugation at 4000 r.p.m. for 20 minutes. The sediment (nuclei and cell debris) was washed once more with several volumes of 0·1 per cent Tween 80, and the wash was added to the soluble components. The nuclei fraction was then deproteinized by successive washing with different concentrations of sucrose solutions (Chauveau, Moule and Rouiller, 1957). In the application of this procedure, the nuclei were suspended in 5·0 ml. of an 0·18 M sucrose-0·0006 M $MgCl_2$ solution, and centrifuged at 500 × *g* for 20 minutes. The sedimented nuclei were next suspended in a 2·3 M sucrose-0·003 M $MgCl_2$ solution, centrifuged for 1 hour at 20,000 × *g*, and the nuclei were washed once more in 0·18 M sucrose-0·0006 M $MgCl_2$ solution at 500 × *g* for 20 minutes. The partially purified nuclei fraction was stored at −20° C. until analyzed. Aliquots of the resulting cell fractions and culture media were precipitated with TCA as follows: One volume of 10 per cent TCA was added to 1 volume of the test fraction, the precipitate was collected on a millipore filter (25 mm. diameter, 0·45 $\mu$ porosity), and the precipitate was washed twice with 1 volume each of 5 per cent TCA. The filters containing the precipitated samples, as well as 0·1–0·2 ml. aliquots of the filtrates were dried overnight at 40° C. in Packard vials. Approximately 15 ml. of scintillation fluid were added to the well-dried samples and the activity was obtained in a Tri-Carb liquid scintillator. Quenching controls were included and the specific activity was expressed as acid precipitable CPM/mg. cell protein or per $\mu$g. of cell DNA. *Separation of soluble cell protein on Sephadex:* The soluble components obtained from normal cells which had been pulse labelled with $H^3$-3,4-BP-caf for 60 minutes (described above) and subsequently cultured for 24 hours, were separated on sephadex G 50 as follows: a dialyzed sample containing 0·5 mg. protein and an activity of 16,000 CPM ($H^3$) was put onto the sephadex column (0·5 × 8 cm.) which had previously been washed with distilled water. The soluble protein fraction(s) were eluted (3 ml./tube/hour) with 1·0 M NaCl. Aliquots of the eluants and controls were analyzed for radioactivity with the liquid Tri-Carb Scintillator.

### RESULTS

In order to study the effect of 3,4-benzopyrene coupled to caffeine in aqueous solution (BP-caf) on the growth of normal or neoplastic cells in culture, it seemed necessary to test first the cyto-toxicity of caffeine itself. Secondary monolayer cultures of normal embryo and tumour cells were exposed to varying concentrations of caffeine dissolved in the complete medium. The results of toxicity of the cells to caffeine, illustrated in Table I, showed that caffeine at certain levels permitted normal cell functions (protein synthesis) with no detectable evidence of cytotoxic damage. The level of caffeine which was demonstrated to possess no toxicity for both normal and tumour cells was found to be 12·0 μg./ml. complete medium or 62·5 μM.

TABLE I.—*The Toxicity Response of Normal and Tumour Cells to Different Concentrations of Caffeine*

| Cell type | Dish size (mm.) | μg. caffeine/ml. complete medium | Cyto-toxicity* 120 hr. | Toxicity response μg. cell protein/dish (hr.) 0 | 24 | 48 | 72 | 120 |
|---|---|---|---|---|---|---|---|---|
| C57Bl embryo | 150 | — | — | 400 | 1100 | nd | 1400 | 1800 |
| | | 97·0 | + | | 760 | ,, | 1300 | 1550 |
| | | 29·0 | — | | 950 | ,, | 1500 | 1800 |
| | | 12·0 | — | | 1150 | ,, | 1500 | 1850 |
| C3H embryo | 50 | — | — | 90 | 250 | 460 | 690 | 980 |
| | | 97·0 | + | | 210 | 400 | 550 | 600 |
| | | 12·0 | — | | 230 | 440 | 570 | 1000 |
| C7 tumour (C3H) | 50 | — | — | 100 | 175 | 252 | 320 | 560 |
| | | 97·0 | + | | 140 | 200 | 150 | 220 |
| | | 12·0 | — | | 176 | 260 | 280 | 580 |

nd = not determined.
* = distinct toxicity response manifested by rounding up, granularity and detachment of cells from the glass surface.

Previous experiments (Alfred, Globerson, Berwald and Prehn, 1964) indicated that normal embryo cell cultures, exposed to paraffin impregnated discs of 3,4-benzopyrene particles, produced greater amounts of total protein per cell than untreated controls. The question whether soluble 3,4-BP complexes would similarly " stimulate " an increase in protein synthesis in the same cell system was analyzed. Total protein values of cell digests of normal cells grown in the presence of complete medium (supplemented by 0·0033 μg. 3,4-BP-caf/ml.) were compared to the protein values of control cultures grown in complete medium (supplemented by 12·0 μg. caffeine/ml.). The results shown in Fig. 1 and 2 demonstrate that low concentrations of benzopyrene *in vitro* caused a rapid increase in protein synthesis (protein per cell) accompanied by an increase in nucleic acid, as compared to control cells grown in medium containing caffeine alone.

The possibility of determining minute quantities of benzopyrene in solution by fluorescence when 3,4-BP-caf complex was mixed with culture medium suggested a measure of the disappearance of this agent from the medium as a function of time. Secondary monolayer cultures were exposed to 3,4-BP-caf in complete medium for varying periods of time. Following these periods of incubation, the culture fluids were removed and centrifuged at 5000 r.p.m. for 15 minutes, and the cells were washed and harvested as described in Materials and Methods, Section 3. The results recorded in Table II demonstrate that the specific fluorescence material disappeared from the medium after 24 hours, but this material could not be detected by fluorescence in the total cell digests. However, sub-

FIG. 1.—The effect of soluble 3,4-benzopyrene (0·0033 μg./ml.) caffeine complex on the growth of normal C57Bl mouse embryo cells. Control cultures contained 12·0 μg./ml. caffeine. Cell number is represented on a logarithmic scale.

FIG. 2.—A comparison between the quantity of the 280 mμ and the 260 mμ absorbing substances formed by 3,4-BP-caffeine (0·0033 μg. BP + 12·0 μg. caffeine/ml.) treated and control (12·0 μg. caffeine/ml.) cultures of C57Bl embryo cells.

TABLE II.—*Fluorescence Analysis* of the Culture Fluids of Normal C57Bl Mouse Embryo and C7 Tumour Cell Cultures Exposed to 3,4-Benzopyrene-caffeine Complex*

| Cell type | Added to complete medium | Fluorescence units† of 3,4-BP in culture medium (hr. culture) 0 | 24 | 48 | 72 | 120 | 192 |
|---|---|---|---|---|---|---|---|
| C57Bl. | caffeine | 2·6 | 1·2 | 3·0 | 3·0 | 2·7 | 3·0 |
| embryo | 3,4-BP-caf | 20·0 | 4·0 | 3·5 | 3·8 | 4·0 | 3·1 |
| C7 | caffeine | 3·0 | 0·9 | 0·9 | 0 | 0 | 0 |
| tumour | 3,4-BP-caf | 20·0 | 1·6 | 1·6 | 3·0 | 1·0 | 1·0 |

* In fluorescence measurements the activation = 390 m$\mu$, and the fluorescence = 410 m$\mu$. The culture medium at 0 hours was supplemented either by 0·01608 $\mu$g. 3,4-BP/ml. or by 58·2 $\mu$g. caffeine/ml.

† Arbitrary units obtained by multiplying the per cent transmission at the above wave lengths by a constant meter multiplication.

sequent experiments using pulse labelling techniques did show that the benzopyrene had been taken up by the individual cells.

Secondary monolayer cultures of normal embryo and tumour cells were pulse labelled with tritiated 3,4-benzopyrene (coupled to 0·004 M caffeine). Cell suspensions in complete medium were seeded into glass dishes and incubated for 3–4 hours under standard conditions. The attached cell sheets were washed and pulse labelled with the tritiated 3,4-BP-caf agent (0·064 $\mu$g./BP ml. Eagle's medium minus serum) for varying minute intervals. Control cultures were treated with Eagle's medium containing the same concentration of caffeine (58·2 $\mu$g./ml.) as the carcinogen treated cells. The pulse labelling fluids were harvested and assayed for the disappearance of $H^3$-3,4-BP as a function of time, and the cell sheets were again washed with Eagle's medium minus serum and reincubated in complete medium for an additional 96 hours. Following incubation, the cells were digested and analyzed for radioactivity of the TCA precipitable fractions. The results of a series of these experiments are summarized in Fig. 3, 4 and 5 in which the relative rates of disappearance of $H^3$-3,4-BP from the pulse labelling fluids of normal and tumour cell cultures are compared. It was found unexpectedly that normal cells showed an uptake of carcinogenic agent (per mg. of cell protein or per $\mu$g. of DNA) of a significantly lower level than did tumour cells, although these tumour cells were reported to be more resistant to cytotoxic damage of 3,4-BP than normal cells (Alfred, Globerson, Berwald and Prehn, 1964). It should be noted that the cell number and cell size of the two types of cultures were same at the time of carcinogen application. Yet the ratio of tumour cell uptake of $H^3$-3,4-BP to that of normal cells was consistently about 10 : 1 as a function of time of exposure (Fig. 3).

Further attempts were then made to establish the probable site of intracellular interaction of $H^3$-3,4-BP and 3 hour old mouse embryo cell cultures, containing 5–8 $\times$ $10^6$ cells/150 mm. dish. The washed cell sheets were pulse labelled for 30 or 60 minute intervals with known concentrations of the $H^3$-3,4-BP-caf complexes. Following labelling the cell sheets were washed as previously described and cultured in complete medium (minus carcinogen) for 24–120 hours. After cultivation the cells were harvested by scraping, washed with PBS (4° C.) by low speed centrifugation and an aliquot digested with 1 volume of 0·1 N NaOH

FIG. 3.—Relative rates of disappearance of $H^3$-3,4-BP from the pulse labelling medium⁺, and uptake by normal and tumour cell cultures. + = the pulse labelling medium containing 0·064 $\mu$g. $H^3$-3,4-BP (in 0·004 M caffeine)/ml. Eagle's medium, minus serum.

FIG. 4.—The binding of $H^3$-3,4-BP by normal and tumour cells in culture. Analysis was made 96 hours following pulse labelling (0–60 min.) and cultivation in complete medium.

FIG. 5.—The binding of $H^3$-3,4-BP by normal embryo and tumour cells in culture/$\mu$g. cell DNA, as a function of time of exposure.

at 4° C. The remaining portion was fractionated into its soluble cell components (sol. C.C.) and nuclei, and the nuclei fraction was digested with 1 volume of 0·1 N NaOH at 37° C. for 15 minutes. All of the cell fractions were precipitated with TCA and assayed for radioactivity. The results of experiments on the binding of $H^3$-3,4-BP are recorded in Table III.

TABLE III.—*The Binding of $H^3$-3,4-BP by Secondary Monolayer Cultures of Normal C57Bl Mouse Embryo Cells*

| Experiment | CPM/ml. $H^3$-3,4-BP (time$_0$) | Exposure $H^3$-3,4-BP (minutes) | Hours culture | Fraction | Total acid ppt. CPM | SP. act. (CPM/mg. cell protein) |
|---|---|---|---|---|---|---|
| I | 900,000 | 60 | 120 | cell digest | 47,000 | 31,000 |
| | | | | sol. C.C. | 132,000 | 33,000 |
| | | | | nuclei | 12,000 | 10,000 |
| II | 250,000 | 30 | 120 | cell digest | 12,000 | 6,000 |
| | | | | sol. C.C. | 45,000 | 4,800 |
| | | | | nuclei | 10,000 | 2,500 |
| III | 840,000 | 60 | 96 | sol. C.C. | 214,000 | 28,500 |
| | | | | nuclei | 37,000 | 6,400 |
| IV | 400,000 | 30 | 24 | sol. C.C. | 365,000 | 30,000 |
| | | | | nuclei | 55,000 | 17,000 |

The specific activities (Sp. act) recorded in Table III show that the soluble fraction, mainly soluble cell protein, contained the majority of the tritium label. When the activity of the soluble fractions of experiments I and II are compared to that of the cell digest fractions, 80–100 per cent of the radioactivity is associated with the soluble cell components. Of interest is the high recovery of activity in the soluble fraction of experiment IV to the relative short period of culture. This finding was reproducible in subsequent experiments and suggested that maximum carcinogen binding took place before actual active cell division began and the protein content per cell was unchanged from time zero.

Fig. 6.—Separation of the soluble protein fraction from a 24 hour culture of normal mouse embryo cells which had been pulse labelled for 60 minutes (3 hours after seeding) with $H^3$-3,4-BP-caf.

Further evidence that the $H^3$-3,4-BP agent was bound to macromolecules of the cells is demonstrated by the results obtained from separation of the soluble cell fractions on sephadex G 50 columns. An 0·5 ml. sample of a dialyzed (72 hours × 3 changes of distilled $H_2O$ at 4° C.) preparation of the sol. cell component fraction from experiment IV (Table III), containing an activity of 16,000 CPM/ 0·5 mg. protein, was put on the equilibrated column. The separation of the radioactively bound soluble cell protein fraction on sephadex is illustrated in Fig. 6.

Small aliquots in triplicate were taken directly from the eluted samples, dried overnight, and assayed for radioactivity. It was demonstrated that the total recovery of tritiated material which was eluted along with 280 m$\mu$ absorbing components was 81·2 per cent in terms of CPM/O.D. unit. It was not determined whether or not the tritiated samples had the same fluorescence spectrum as the original agent used in carcinogen-cell interactions.

### DISCUSSION

Chemical carcinogens in suspension were shown to exert *in vitro* a cytotoxic effect on normal cells, whereas tumour cells, tested under comparable conditions, were found to be resistant towards this effect (Alfred, Globerson, Berwald and Prehn, 1964). To analyze further the cellular and molecular basis of the interaction between carcinogenic hydrocarbons and cells *in vitro*, benzopyrene was applied in a soluble form, complexed with caffeine. The application of such a system, once again demonstrated the relative resistance of tumour cells to the cytotoxic effect of the carcinogen. Theoretically, there are two alternative mechanisms which may account for the resistance of tumour cells to the cytotoxic effects of the carcinogen : (1) Resistance due to the impermeability of the tumour cells to the carcinogen. (2) Resistance, in spite of the penetration of the carcinogen to the tumour cell. The application of $H^3$-3,4-BP has clearly demonstrated that the carcinogen is, in fact, taken up by the tumour cells. Furthermore, uptake of the carcinogen by these cells was by a factor of 10 greater than its uptake by normal cells. The resistance under these conditions could be explained if in the tumour cells a deletion has taken place of those proteins which bind the carcinogens in the normal cells. Indeed, the concept of protein deletion has been the basis of the mechanism of carcinogenesis proposed by the Millers (1953) and Abell and Heidelberger (1962) have further demonstrated that tumour cells have, in fact, lost proteins, which in normal cells bind hydrocarbon carcinogens. In the present study, however, we found that the carcinogen label was bound to the aqueous soluble fraction (TCA precipitable) of the tumour cells. This may not necessarily mean that in the tumour cells tested no deletion of the proteins which interact with the carcinogen has taken place. It only indicates that definitely not *all* proteins which bind the carcinogen in the normal cell have been deleted. A further analysis of the protein fractions of tumour cells is required before it is possible to establish whether or not any proteins specifically involved in chemical carcinogen binding have been deleted.

Among the manifestations of cell carcinogen interaction which may be relevant to the neoplastic transformation, the increased net synthesis of proteins, per surviving normal cell, should be noted.

The experiments designed to show the site of binding between $H^3$-3,4-BP and normal cells in culture demonstrated that the soluble cell fraction was predominantly involved. Upon separation of the soluble cell components of normal treated cells on sephadex columns, the soluble protein fraction contained more than 80 per cent of the total radioactivity originally bound. It is therefore concluded from these findings that the soluble cell protein fraction is the probable principal site of binding of 3,4-BP in soluble form, and that both normal and tumour cells *in vitro* possess the ability to take up significant amounts of this carcinogen. Studies now in progress are directed toward the isolation of the cellular components involved in chemical carcinogen interaction and an analysis of the nature of binding by neoplastic cells. The culture system described in which the cells were treated with low concentrations of aqueous solutions of hydrocarbon carcinogen, may possibly be a useful tool in a defined study of the alterations in growth control mechanisms in mammalian cells.

SUMMARY

A study was made of the uptake of benzopurene by normal and tumour cells *in vitro*, using 3,4-benzopyrene in aqueous solution (i.e. coupled to caffeine). Normal mouse embryo cells, which survived the cytotoxic damage caused by the carcinogen, were found to synthesize a greater quantity of protein per cell than do untreated controls. Both normal and tumour cells removed 3,4-benzopyrene from the medium during the first 24 hours of culture, as measured by specific fluorescence. However, when normal and tumour cells, under identical culture conditions, were exposed to low concentrations of tritiated 3,4-BP (0·064 $\mu$g./ml.) soluble complexes, tumour cells took up the labelled carcinogen to a 10-fold greater concentration than did normal cells. The maximum uptake of $H^3$-3,4-BP/mg. cell protein for both cell types took place between 10–30 minutes of exposure, although the relative rates were different.

Separation of the soluble components from the nuclei of $H^3$-3,4-BP treated normal embryo cells, by disruption with 0·1 per cent Tween 80 and subsequent centrifugation, showed the radioactivity to be predominantly associated with the soluble fraction. Approximately 81·2 per cent of the radioactivity bound to the soluble fraction was recovered with the soluble cell protein by gel filtration on sephadex G 50. The differential capacity of normal and neoplastic cells *in vitro* to bind soluble complexes of tritiated 3,4-benzopyrene was discussed.

The author wishes to express gratitude to Mrs. V. A. Parsegian for excellent technical assistance. The guidance and suggestions of Professor M. Feldman in the execution of these experiments and in the preparation of this manuscript are sincerely acknowledged.

This work was supported by a Post-Doctorate Fellowship (C-9717), National Cancer Institute, NIH, Bethesda, Maryland.

### REFERENCES

Abell, C. W. and Heidelberger, C.—(1962) *Cancer Res.*, **22**, 931.

Alfred, L. J., Globerson, A., Berwald, Y. and Prehn, R. T.—(1964) *Brit. J. Cancer*, **18**, 159.

Berenblum, I. and Schoental, R.—(1946) *J. chem. Soc.*, p. 1017.

Boyland, E. and Green, B.—(1962) *Brit. J. Cancer*, **16**, 347.

Burton, K.—(1956) *Biochem. J.*, **62**, 315.

Chauveau, J., Moule, Y. and Rouiller, C.—(1957) *Bull. Soc. Chim. biol., Paris*, **39**, 1521.

Fisher, H. W. and Harris, H.—(1962) *Proc. Roy. Soc.*, B., **156**, 521.

Heidelberger, C. and Davenport, G. R.—(1961) *Acta Un. int. Cancr*, **17**, 55.

Lowry, G. H., Rosebrough, N. J., Farr, A. L. and Randall, R. J.—(1951) *J. biol. Chem.*, **193**, 265.

Miller, J. A. and Miller, E. C.—(1953) *Advanc. Cancer Res.*, **1**, 547.

Pitot, H. C. and Heidelberger, C.—(1963) *Cancer Res.*, **23**, 1694.

Starikova, V. B. and Vasiliev, Y. M.—(1963) *Acta Un. int. Cancr*, **19**, 620.

Weil-Malherbe, H.—(1946) *Biochem. J.*, **40**, 351.